# Origin of reduced magnetization and domain formation in small magnetite nanoparticles

**DOI:** 10.1038/srep45997

**Published:** 2017-04-10

**Authors:** Zlatko Nedelkoski, Demie Kepaptsoglou, Leonardo Lari, Tianlong Wen, Ryan A. Booth, Samuel D. Oberdick, Pedro L. Galindo, Quentin M. Ramasse, Richard F. L. Evans, Sara Majetich, Vlado K. Lazarov

**Affiliations:** 1 Department of Physics, University of York, Heslington, York, YO10 5DD, UK; 2 SuperSTEM, Sci-Tech Daresbury Campus, Daresbury, WA4 4AD, UK; 3 Physics Department, Carnegie Mellon University, Pittsburgh, Pennsylvania 15213, USA; 4 State Key Laboratory of Electronic Thin Films and Integrated Devices, University of Electronic Science and Technology of China, Chengdu, China; 5 Department of Computer Science and Engineering, Universidad de Cádiz, 11510 Puerto Real, Spain

## Abstract

The structural, chemical, and magnetic properties of magnetite nanoparticles are compared. Aberration corrected scanning transmission electron microscopy reveals the prevalence of antiphase boundaries in nanoparticles that have significantly reduced magnetization, relative to the bulk. Atomistic magnetic modelling of nanoparticles with and without these defects reveals the origin of the reduced moment. Strong antiferromagnetic interactions across antiphase boundaries support multiple magnetic domains even in particles as small as 12–14 nm.

Magnetic nanoparticles (NPs) are expected to be single magnetic domain below a critical size, typically on the order of the domain wall width for the corresponding bulk material. Single domain particles should have the maximum magnetic moment per volume, which is desirable for their use in ferrofluids^1^,^2^, magnetic separation^3^, contrast agents^4^,^5^ for magnetic resonance imaging and magnetic hyperthermia^6^,^7^, all of which use iron oxide NPs. However, many researchers observe reduced magnetization, relative to that of the bulk. The reduction has been attributed to surface spin disorder^8^,^9^ or to variations in crystallinity, as in the case of low temperature aqueous preparation methods^10^. However, increasing crystallinity and reducing surface roughness does not necessarily solve this problem^11^,^12^. Indeed, in this work we demonstrate that even high quality magnetite NPs can have dramatic differences in their magnetic properties. High resolution electron microscopy is used to identify subtle differences in three kinds of NPs, and atomistic spin calculations are used to reveal the origin of the reduced magnetization and its anomalous temperature dependence.

By a combination of measurements and modelling we demonstrate that antiphase domain boundaries in NPs can greatly reduce their magnetic moment. APB defects are seen in substrate supported thin film growth of magnetite^13^,^14^,^15^, and also in core-shell NPs^16^. Here we demonstrate that the strong antiferromagnetic super-exchange interactions across the APBs significantly decrease the magnetisation of the NPs due to formation of multiple magnetic domains even in NPs below 15 nanometres.

While the detection of APBs requires sophisticated atomic resolution electron microscopy, we show that besides the reduced M_s_, zero field cooled magnetization measurements can be used to easily identify types of NPs with a high density of these defects, and therefore lower magnetization compared with that of single crystal NPs.

## Results and Discussion

**Figure 1:**
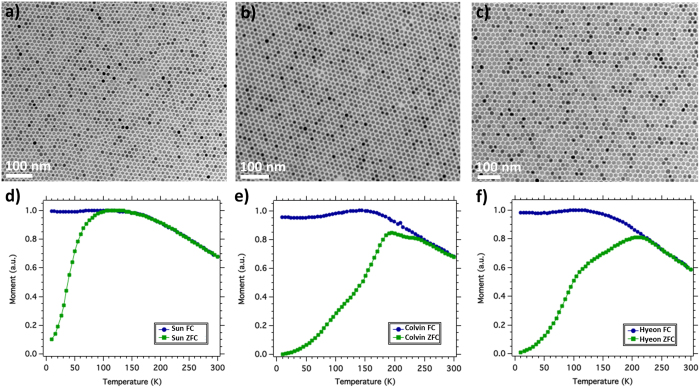
Conventional TEM images of (**a**) Sun, (**b**) Colvin, (**c**) Hyeon nanoparticles. Zero Field Cooled (ZFC) and Field Cooled (FC) magnetization curves for (**d**) Sun, (**e**) Colvin, (**f**) Hyeon nanoparticles.

The experiments compared 12–14 nm diameter iron oxide nanoparticles synthesized by three well-established methods that were pioneered by the groups of Sun^17^, Colvin^18^ and Hyeon^19^. Hereafter, they will be referred to as Sun, Colvin and Hyeon NPs. All three synthesis methods involve high temperature inert atmosphere decomposition in organic solvents, leading to monodisperse, highly crystalline, spherical particles coated with surfactants. Large field of view electron microscopy images for all three sets of NPs are presented in Fig. 1a–c and Supplementary Fig. S1. The size distribution of the three samples is similar: Sun 12.3 ± 2.9 nm, Colvin 13.7 ± 1.6 nm and Hyeon 14.2 ± 2.0 nm.

The structural and chemical composition of the particles was characterized by a combination of selected area electron diffraction (SAED) and electron energy loss spectroscopy (EELS). In some syntheses of magnetite (Fe_3_O_4_) other phases are formed initially, then transformed through oxidation^16^. In contrast, as shown in Supplementary Fig. S2, all sets of the studied NPs in this work are single phase magnetite NPs. For more detailed chemical information, EELS measurements were performed in an aberration corrected scanning transmission electron microscope (STEM). The small size of the aberration corrected electron probe (<1 Å in the conditions used for this experiment) reveals the local chemical composition within the nanoparticles. Furthermore, comparison of O *K* and Fe *L*_2,3_ edge fine structures averaged over selected NPs with those acquired from a known ‘bulk’ Fe_3_O_4_ sample, acquired under identical optical conditions (Supplementary Fig. S3) confirms that the chemistry of all three sets nanoparticles is Fe_3_O_4_ like (Supplementary EELS discussion). In addition, atomically-resolved 2D EELS measurements for all three sets NPs (Supplementary Figs S4, S5 and S6) consistently showed no change in the Fe *L*_*2*,*3*_ white-line intensity ratios across the observed NPs. This confirms the chemical uniformity of the particles and the absence of a core-shell structure.

In contrast to the apparent structural and chemical uniformity of the three sets of nanoparticles, as determined by TEM, SAED and EELS, the magnetic properties are strikingly different. The Sun particles have a specific saturation magnetization of 81 ± 12 emu/g at 10 K (71 ± 1 emu/g at 300 K), values that are roughly 10% below that of the bulk. In comparison, the values for the Colvin particles were 37 ± 1 emu/g at 10 K (36 ± 1 emu/g at 300 K), and for the Hyeon particles 39 ± 4 emu/g at 10 K (40 ± 4 emu/g at 300 K). These Colvin and Hyeon particles have approximately half the magnetization of the Sun particles of comparable size. When hysteresis loops were measured after cooling in a large magnetic field, no exchange bias was observed.

Further details about the magnetic behaviour of the NPs are revealed by the zero field cooled (ZFC) and field cooled (FC) magnetization curves (Fig. 1d–f). The ZFC curve of the Sun particles shows a peak at 110–120 K, and a gradual decay at higher temperature. The Colvin particles also show a shoulder at ~ 110–120 K, plus a peak at 180 K and another feature near 200 K. The Hyeon particles have a shoulder near 110 K and a peak slightly above 200 K. The peak in the ZFC magnetization curve is associated with the blocking temperature *T*_B_ of the nanoparticles, which is proportional to the anisotropy: *KV* ~ 25 k_B_*T*_B_, where *K* is the anisotropy energy density, *V* is the particle volume and k_B_ is the Boltzmann constant. With this interpretation, the Colvin and Hyeon particles would have higher *K* than the Sun particles, yet they have lower magnetization. ZFC curves with anomalous features like those in Fig. 1e,f have been reported for NPs made by many other methods including aqueous co-precipitation^20^. ZFC curves like that of Fig. 1d have been reported for larger aqueous method particles^21^.

**Figure 2:**
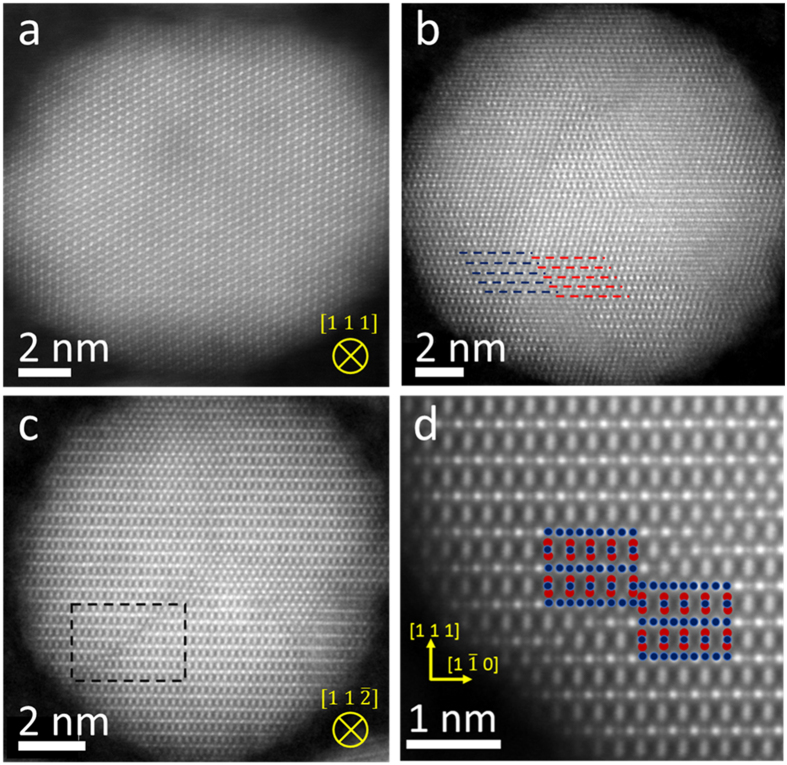
Atomically resolved HAADF STEM images of representative (**a**) Sun NP viewed along the [111] zone axis, (**b**) Colvin NP viewed along the [114] zone axis. (**c**) Hyeon NP along the [11-2] zone axis, obtained by rigid registration of a stack of images of the same area recorded in quick succession (resulting in high signal-to-noise and precision in the image). Dashed lines in (**b**) and (**c**) indicate the presence of the structural defects. (**d**) Magnified view of the dashed area shown in (**c**) with overlaid structural model emphasizing the defect region.

The puzzling magnetic behaviour in the ZFC curves cannot be explained by the conventional TEM and SAED observations alone, but requires deeper structural analysis. STEM imaging was used to determine the atomic structure and chemistry of selected nanoparticles, and to identify structural characteristics typical to each preparation method. The contrast dependence of high angle annular dark field (HAADF) imaging on the atomic number *Z* as ~*Z*^*1.7*^ enables direct identification of both iron tetrahedral (Fe_A_) and octahedral (Fe_B_) atomic columns in the NPs. Figure 2 shows atomically-resolved HAADF images of representative Sun, Colvin and Hyeon NPs. In all three cases the bulk-like magnetite structural ordering extends to the particle surfaces, and no core-shell structure is observed.

However, upon closer inspection, the HAADF images reveal certain structural characteristics that may be related to the observed differences in magnetic behavior. The Sun nanoparticles, such as that shown in Fig. 2a are predominantly spherical with no strain (Supplementary Fig. S7) or disorder across the entire particle, as in metallic NPs^22^. In contrast to the Sun NPs, structural defects are observed in both Colvin and Hyeon NPs. The prevalence of structural defects in Colvin and Hyeon NPs and absence of defects in Sun NPs is demonstrated by dark-field TEM images covering hundreds of NPs (Supplementary Figs S8, S9 and S10). In addition, the presence/absence of structural defects is illustrated in the atomic resolution HAADF STEM images for the three sets, presented in Supplementary Figs S11, S12 and S13. Figure 2b,c shows that the translational symmetry is broken and structural domains are formed. Most of the observed structural defects are antiphase domain boundaries (APBs), which can be described by fractional unit cells shift of ¼*a*_0_[110]. In Fig. 2c the selected region shows the atomic arrangement of an APB with ¼a_0_ <110> viewed along the [11-2] zone axis. The mismatch across the boundary (e.g. between octahedral planes across the boundary) is outlined. Figure 2d shows the break in the structural symmetry of the cationic (111) plane by a crystallographic shift of the octahedral atomic planes by 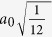, imaged along the [11-2] viewing direction.

**Figure 3:**
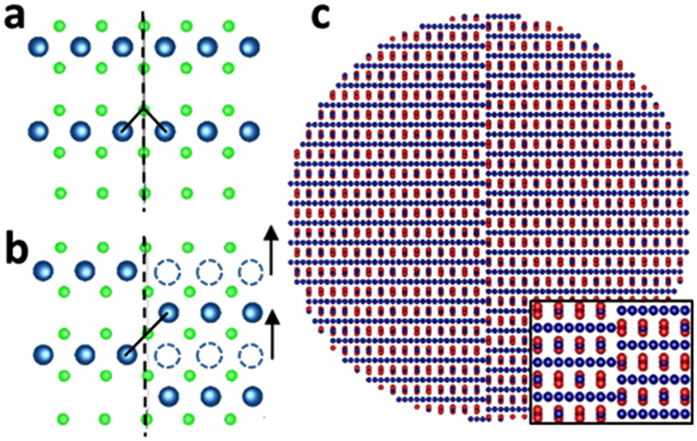
(**a**) [001] view of a (001) Fe_B_–O lattice plane in bulk magnetite. (**b**) The presence of a ¼a_0_ <110> APB (dashed line) leads to shifted right hand side for ¼a_0_ <110> (in-plane, arrows represent the shift vector), changing the Fe_B_-O-Fe_B_ angle from 90° as shown in (**a**) to 180°. The oxygen sub-lattice is invariant under the shift. (**c**) 10 nm nanoparticle model with single ¼a_0_ <110> APB shown along the [11-2] zone axis. Tetrahedral Fe_A_ atoms are coloured in red, octahedral Fe_B_ in blue and oxygen atoms (not shown in (**c**)) in green. Drawing of the structural models was generated in VESTA^39^ software.

APBs, extensively studied in magnetite thin films^13^,^23^,^24^,^25^,^26^,^27^, are correlated with anomalous properties such as very high magnetic saturation fields^23^ and negative magnetoresistance^15^,^28^. However, no direct evidence of APBs in single crystal magnetite NPs has been demonstrated. Recent work on the formation energy of highly stable APB defects^29^ could explain why APBs are stable even in NPs. A lattice vector shift of ¼ *a*_0_ <110> creates an APB (Fig. 3a,b) with a very low formation energy (0.1 J/m^2^). Figure 3b illustrates how the 90° (Fig. 3a) Fe_B_-O-Fe_B_ bulk-like ferromagnetic super-exchange interaction is changed to 180° antiferromagnetic interaction due to the lattice shift caused by the APB. APBs with different lattice shift vectors result in similarly increased AFM interactions. Here we consider only the APBs with ¼ a_0_ <110> shift for two reasons: a) these APBs are experimentally observed in the studied NPs, and b) the formation energies of other type APBs are an order of magnitude larger, and are therefore much less likely to form in NPs.

These experimental findings provide a basis for an atomic level understanding of the different magnetic behavior, based on the strong correlation between the macroscopically measured magnetic properties of NPs and the presence of the APB defects observed by atomic level imaging. The magnetic properties of magnetite are determined by the short-range Fe-O-Fe super-exchange interactions. The dominant super-exchange interaction in Fe_3_O_4_ between tetrahedral Fe_A_ and octahedral Fe_B_ sublattices results in their antiferromagnetic alignment^30^,^31^. Since there are twice as many Fe atoms on B sites as on A sites, this gives an overall net magnetisation of 4 μ_B_ per formula unit of Fe_3_O_4_. The super-exchange interactions depend strongly on the angle and the length of the Fe-O-Fe bonds^32^. When the angle is 90° (the case of Fe_B_-O-Fe_B_ bonds) the super-exchange interaction is ferromagnetic. As the bond angle increases the super-exchange becomes antiferromagnetic (AFM), as in the case of 125° Fe_A_-O-Fe_B_ bond that is the dominant super-exchange interaction in magnetite. Any further increase in angle, increases the strength of AFM interaction, which reaches a maximum value for 180° bonds^33^. Across the APB the Fe-O-Fe bond angles are distorted which significantly impacts the magnetic properties.

**Figure 4:**
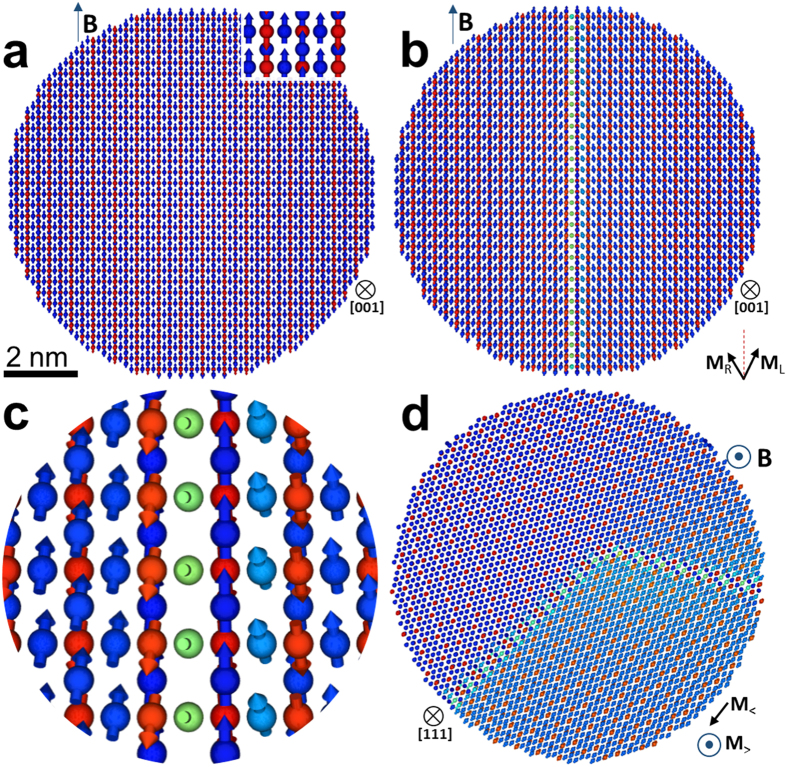
Spin configurations at 5T. (**a**) [001] zone axis of a 10 nm nanoparticle without APBs, which has a single magnetic domain. (**b**) [001] zone axis of a 10 nm nanoparticle with a ¼a_0_ <110> APB, which leads to a coincident magnetic domain wall. The net magnetisations of the left and right part of the NP are labelled as M_R_ and M_L_, respectively. (**c**) Magnified view of (**b**) near the APB plane showing the canting of the spins at the boundary. (**d**) 10 nm model with two non-coplanar APBs on <1 1-2> type planes, with net magnetization M_>_ (along the field) and M_<_ (in-plane) for the upper and lower part respectively. The colour coding is in RGB fashion where the function which determines the colour is dependent only on the projection of the spin direction along the applied field. Two limiting cases are pure blue (R = 0; G = 0; B = 1) for spins aligned along the field and pure red colour for spins in opposite direction. Net magnetization direction (determined by the Fe_B_ spins) in each of the domains helps identify the Fe_B_ sites.

Atomistic spin calculations on model particles (with and without APBs) provide insight into the effect of APBs on NP magnetic moment. Using VAMPIRE software^34^ we calculated the spin configurations at magnetic saturation for various model NPs and compared the results to same size NP without APBs (Fig. 4a). Figure 3c shows the atomic geometry of the simplest model, a spherical nanoparticle 10 nm in diameter, with a ¼a_0_ <110> APB at the center of the NP. The calculations for this model NP show that there is a reduction in the saturation magnetization by 26% compared to the same size NP without the structural defect (i.e. magnetization value of ~66 emu/g). Furthermore, looking at the spin configuration snapshots (Fig. 4a,b) one can see the formation of two magnetic domains due to the presence of the strong 180°antiferromagnetic bonds across the boundary (Fig. 4c). Within a domain, the Fe_A_ and Fe_B_ spins are antiparallel, as expected in bulk magnetite. However, across the APB, the Fe_B_ spins are canted because of the change in Fe_B_-O-Fe_B_ superexchange coupling. In Fig. 4b, where the NP has an APB across its center, the magnetization of the left (M_L_) and right (M_R_) sides of the particle are not parallel, even at 5T. The APB reduces the magnetic moment, relative to a defect-free NP.

This simple model demonstrates the effect of the APBs on the NP magnetic moment, but more complex NPs with multiple APBs, faceting, and surface anisotropy were also studied. When the number of coplanar APBs and their relative positions were varied, the magnetization reduction in all modeled NPs is determined by the number of 180° Fe_B_-O-Fe_B_ bonds per unit volume. In Fig. 4d we consider two non-coplanar APBs with boundaries on <11-2> type planes, a model inspired by the APBs geometry observed in the Hyeon NP shown in Fig. 2c,d. The predicted decrease in the saturation magnetization for this model NP is ~34% (i.e. the model nanoparticle has magnetization of ~59 emu/g), a result quantitatively closer to the experimental data. Given the small nanoparticle size, surface effects can affect the magnetic properties. Nominally spherical particles can develop faceted surfaces either directly during synthesis or over time via Ostwald ripening. Supplementary Fig. S14 shows that this has little impact on the spin configuration and therefore the particle moment. Surface anisotropy can have important effects in NPs^30^,^35^. However, the NPs here are coated with organic surfactants with weak spin-orbit coupling, hence the surface anisotropy contributions are negligible compared to the strong exchange interaction across the APB defects. Even when large surface anisotropy was assumed, as shown in Supplementary Fig. S15 there was minimal distortion of the spin configuration and the magnetic moment was reduced by only 1%.

## Conclusions

Nanoparticles prepared by the Sun, Colvin and Hyeon methods have similar structure and chemical composition, as confirmed by conventional TEM imaging, SAED, and EELS. However, their magnetic properties are very different. Sun NPs had close to bulk like specific magnetization while Hyeon and Colvin NPs showed less than half of the specific magnetization of bulk Fe_3_O_4_. This anomalous magnetic behavior was correlated with the absence or presence of antiphase domain boundary structural defects. Atomic resolution STEM-HAADF imaging clearly shows the presence of abundant APB defects in the Colvin and Hyeon but not the Sun NPs. The dominant APB occurring in Colvin and Hyeon NPs is due to a ¼ a_0_ <110> lattice vector shift. Due to the very low energy formation of this type boundary they can form under the growth conditions of the Colvin and Hyeon methods. The density of APBs is directly correlated to the number of antiferromagnetic bonds that ultimately lead to the reduced magnetization. Atomistic spin calculations show that the magnetization reduction is greater when more antiferromagnetic bonds are created across the boundary planes. The strong character of the super-exchange interactions is responsible for the multi-domain magnetic behaviour of the Fe_3_O_4_ NPs, a unique property not expected in metallic magnetic-NPs.

In summary, the antiphase boundary structural defects create high angle antiferromagnetic bonds that reduce the magnetization in crystalline magnetite nanoparticles. Magnetic nanoparticles predicted to be mono-domain based on their size alone may in fact contain multiple magnetic domains. Because of their stability, APBs reduce the particle magnetic moment unless extremely large fields are applied. For applications where the magnitude of the particle magnetization is critical and the applied fields are modest, NPs with a low density of APBs are desirable. ZFC magnetization measurements are a convenient way to screen nanoparticles and optimize preparation methods in order to minimize these magnetization-reducing defects.

## Methods

All samples were prepared using Schlenk line techniques, with inert atmosphere decomposition in high boiling point organic solvents. There were slightly different precursors, surfactants, and solvents. In all cases a single growth stage was used to obtain the final particle size. The Sun method^17^ uses Fe acetyl acetonate as a precursor and a combination of oleic acid and oleyl amine surfactants, in benzyl ether. The Colvin method^18^ uses an Fe oxyhydroxide precursor and oleic acid, in 1-octadecene. The Hyeon method^19^ uses an Fe oleate precursor and oleic acid, also in 1-octadecene. Transmission electron microscopy (TEM) was used to assess the size distribution of the particles and selected area electron diffraction (SAED) was used for preliminary phase identification. Dense assemblies of the particles were characterized by superconducting quantum interference device (SQUID) magnetometry to determine the saturation magnetization at 10 K and 300 K, at fields up to 5 T. Zero field cooled (ZFC) magnetization curves were measured after cooling to 10 K, turning on a small field (0.01 T), and measuring the magnetization as the temperature was increased up to 300 K. For field cooled (FC) magnetization curves, the magnetization was recorded as the samples were cooled from 300 K to 10 K in 0.01 T field.

To quantify the specific saturation magnetization, elemental analysis was used to determine the amount of iron. Here the magnetic moment at 5 T of a fixed volume of nanoparticle dispersion in toluene was measured. Atomic absorption measurements were made on the same samples over a series of dilutions, and comparison with a Fe calibration standard was used to determine the molar concentration of iron in the undiluted sample. The numerical values assumed that the iron was in the form of Fe_3_O_4_ and that the particles had the same density as that of bulk magnetite.

Structural characterisation has been performed by transmission electron microscopy (TEM) and Selected Area Diffraction (SAED) using a JEOL 2000 EX and a double aberration corrected JEOL JEM-2200FS, both operated at 200 kV.

Scanning transmission electron microscopy imaging and electron energy loss spectroscopy were performed in a Nion UltraSTEM100^TM^ equipped with a Gatan Enfina spectrometer. The microscope was operated at 100 kV, with a convergence angle of 30 mrad; at these optical conditions the electron probe size is determined to be 0.9 Å; the inner detector angle for HAADF STEM imaging was 76 mrad. The native energy spread of the electron beam for EELS measurements was 0.3 eV; with the spectrometer dispersion set at 0.2 eV/channel, this yielded an effective energy resolution of 0.6 eV. The EELS collection angle was 31 mrad. For reading clarity the atomically resolved spectra presented in Supplementary Figs S4, S5 and S6 were de-noised by principal component analysis using the CiMe^−^ plugin^36^ for Gatan’s Digital Micrograph 2.3 software suite.

The spin dynamics of the magnetite nanoparticles containing APBs were simulated using VAMPIRE software package^34^. The energetics of the system is described by a Heisenberg spin Hamiltonian of the form:


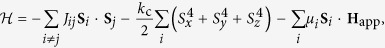


where *J*_*ij*_ is the Heisenberg exchange interaction between neighbouring spins *i* and *j*, **S**_*i*_ and **S**_*j*_ are the local spin unit vectors on sites *i* and *j* respectively, *k*_c_ is the local cubic anisotropy constant, *μ*_*i*_ is the local atomic spin moment on each site *i* and **H**_app_ is the external applied field vector. The demagnetizing field is included in the simulations using the macrocell method^34^ where the atomic spin configuration is spatially averaged over the macrocell. A macrocell size of 2 nm was used in the calculations. A cubic magnetocrystalline anisotropy constant of 3.2·10^−25^ J/atom is included in the simulations. We note that although local cubic anisotropy is included in the simulation, this is relatively weak and has no appreciable effect on the total magnetization of the particle, which is dominated by the exchange interactions. Between Fe_A_ and Fe_B_ sites of magnetite there are corresponding exchange interactions: *J*_AA_ = −0.11 meV, *J*_BB_ = +0.63 meV and *J*_AB_ = −2.92 meV determined from ab-initio calculations^37^. At the APB, the 180° super exchange bonds lead to an enhanced antiferromagnetic exchange interactions with *J*_180_ = −8.9 meV, which was extrapolated from the known bulk exchange interactions using ~cos^2^(*θ*) relationship^38^. All the calculations were performed at temperature set to 0 K in order to show the ground state spin configuration. At higher temperature, spin fluctuations are present within domains and at the surface, but the domain structures remain the same. The simulations focused on spin configurations for NPs at saturation fields (5 T). Details of lower field hysteresis require a full treatment of the local anisotropy introduced by the APB, and are the subject of future work.

### Data Availability

All data created during this research are available by request from the University of York Data Catalogue https://dx.doi.org/10.15124/249cbf0c-8e88-426b-b3ba-53d490e027ed.

## Additional Information

**How to cite this article:** Nedelkoski, Z. *et al*. Origin of reduced magnetization and domain formation in small magnetite nanoparticles. *Sci. Rep.* **7**, 45997; doi: 10.1038/srep45997 (2017).

**Publisher's note:** Springer Nature remains neutral with regard to jurisdictional claims in published maps and institutional affiliations.

## Supplementary Material

Supplementary Information

## References

[b1] Berger P. . Preparation and Properties of an Aqueous Ferrofluid. Journal of Chemical Education 76, 943 (1999).

[b2] Raj K., Moskowitz B. & Casciari R. Advances in ferrofluid technology. Journal of Magnetism and Magnetic Materials 149, 174 (1995).

[b3] Gupta A. K. & Gupta M. Synthesis and surface engineering of iron oxide nanoparticles for biomedical applications. Biomaterials 26, 3995 (2005). 15626447 10.1016/j.biomaterials.2004.10.012

[b4] Laurent S. . Magnetic iron oxide nanoparticles: synthesis, stabilization, vectorization, physicochemical characterizations, and biological applications. Chem Rev 108, 2064 (2008). 18543879 10.1021/cr068445e

[b5] Pereira C., Pereira A. M., Rocha M., Freire C. & Geraldes C. F. G. C. Architectured design of superparamagnetic Fe_3_O_4_ nanoparticles for application as MRI contrast agents: mastering size and magnetism for enhanced relaxivity. Journal of Materials Chemistry B (2015). 10.1039/c5tb00789e 32262745

[b6] Thiesen B. & Jordan A. Clinical applications of magnetic nanoparticles for hyperthermia. International journal of hyperthermia: the official journal of European Society for Hyperthermic Oncology, North American Hyperthermia Group 24, 467 (2008). 10.1080/02656730802104757 18608593

[b7] Pankhurst Q. A., Thanh N. T. K., Jones S. K. & Dobson J. Progress in applications of magnetic nanoparticles in biomedicine. Journal of Physics D: Applied Physics 42, 224001 (2009).

[b8] Pratt A. . Enhanced oxidation of nanoparticles through strain-mediated ionic transport. Nat Mater 13, 26 (2014). 24185757 10.1038/nmat3785

[b9] Kodama R. H., Berkowitz A. E., McNiff J. E. J. & Foner S. Surface Spin Disorder in NiFe_2_O_4_ Nanoparticles. Phys Rev Lett 77, 394 (1996). 10062440 10.1103/PhysRevLett.77.394

[b10] Massart R. Preparation of aqueous magnetic liquids in alkaline and acidic media. Magnetics, IEEE Transactions on 17, 1247 (1981).

[b11] Krycka K. L. . Origin of Surface Canting within Fe_3_O_4_ Nanoparticles. Phys Rev Lett 113, 147203 (2014). 25325655 10.1103/PhysRevLett.113.147203

[b12] Salafranca J. . Surfactant Organic Molecules Restore Magnetism in Metal-Oxide Nanoparticle Surfaces. Nano Letters 12, 2499 (2012). 22497711 10.1021/nl300665z

[b13] Gilks D. . Origin of anomalous magnetite properties in crystallographic matched heterostructures: Fe_3_O_4_(111)/MgAl_2_O_4_ (111). Journal of Physics: Condensed Matter 25, 485004 (2013). 24177186 10.1088/0953-8984/25/48/485004

[b14] Eerenstein W., Palstra T. T. M., Hibma T. & Celotto S. Origin of the increased resistivity in epitaxial Fe_3_O_4_ films. Physical Review B 66, 201101 (2002).

[b15] Margulies D. T. . Origin of the Anomalous Magnetic Behavior in Single Crystal Fe_3_O_4_ Films. Phys Rev Lett 79, 5162 (1997).

[b16] Wetterskog E., Tai C.-W., Grins J., Bergström L. & Salazar-Alvarez G. Anomalous Magnetic Properties of Nanoparticles Arising from Defect Structures: Topotaxial Oxidation of Fe_1–x_O|Fe_3−δ_O_4_ Core|Shell Nanocubes to Single-Phase Particles. ACS Nano 7, 7132 (2013). 23899269 10.1021/nn402487q

[b17] Sun S. . Monodisperse MFe_2_O_4_ (M = Fe, Co, Mn) Nanoparticles. J Am Chem Soc 126, 273 (2004). 14709092 10.1021/ja0380852

[b18] Yu W. W., Falkner J. C., Yavuz C. T. & Colvin V. L. Synthesis of monodisperse iron oxide nanocrystals by thermal decomposition of iron carboxylate salts. Chemical Communications 2306 (2004). 10.1039/b409601k 15489993

[b19] Park J. . Ultra-large-scale syntheses of monodisperse nanocrystals. Nat Mater 3, 891 (2004). 15568032 10.1038/nmat1251

[b20] Daou T. J. . Hydrothermal Synthesis of Monodisperse Magnetite Nanoparticles. Chemistry of Materials 18, 4399 (2006).

[b21] Batlle X. . Magnetic nanoparticles with bulklike properties (invited). J Appl Phys 109, 07B524 (2011).

[b22] Kovács A. . Direct Observation of a Surface Induced Disordering Process in Magnetic Nanoparticles. Phys Rev Lett 103, 115703 (2009). 19792385 10.1103/PhysRevLett.103.115703

[b23] Margulies D. T. . Anomalous moment and anisotropy behavior in Fe_3_O_4_ films. Phys. Rev. B 53, 9175 (1996). 10.1103/physrevb.53.9175 9982420

[b24] Hibma T. . Anti-phase domains and magnetism in epitaxial magnetite layers. J Appl Phys 85, 5291 (1999).

[b25] Luysberg M., Sofin R. G. S., Arora S. K. & Shvets I. V. Strain relaxation in Fe_3_O_4_/MgAl_2_O_4_ heterostructures: Mechanism for formation of antiphase boundaries in an epitaxial system with identical symmetries of film and substrate. Physical Review B 80, 024111 (2009).

[b26] Eerenstein W., Palstra T. T. M., Saxena S. S. & Hibma T. Spin-Polarized Transport across Sharp Antiferromagnetic Boundaries. Phys Rev Lett 88, 247204 (2002). 12059330 10.1103/PhysRevLett.88.247204

[b27] Eerenstein W., Palstra T. T. M. & Hibma T. Spin-valve behaviour of anti-ferromagnetic boundaries in ultrathin magnetite films. Thin Solid Films 400, 90 (2001).

[b28] Eerenstein W., Kalev L., Niesen L., Palstra T. T. M. & Hibma T. Magneto-resistance and superparamagnetism in magnetite films on MgO and MgAl_2_O_4_. Journal of Magnetism and Magnetic Materials 258–259, 73 (2003).

[b29] McKenna K. P. . Atomic-scale structure and properties of highly stable antiphase boundary defects in Fe_3_O_4_. Nat Commun 5, (2014). 10.1038/ncomms6740 PMC4275585 25494005

[b30] Mazo-Zuluaga J., Restrepo J. & Mejía-López J. Surface anisotropy of a Fe_3_O_4_ nanoparticle: A simulation approach. Physica B: Condensed Matter 398, 187 (2007).

[b31] De Grave E., Persoons R. M., Vandenberghe R. E. & de Bakker P. M. Mossbauer study of the high-temperature phase of Co-substituted magnetites, Co_x_Fe_3-x_O_4_. I. x < = 0.04. Physical review B, Condensed matter 47, 5881 (1993). 10.1103/physrevb.47.5881 10004537

[b32] Goodenough J. B. & Loeb A. L. Theory of Ionic Ordering, Crystal Distortion, and Magnetic Exchange Due to Covalent Forces in Spinels. Phys Rev 98, 391 (1955).

[b33] Robinson D. W. Magnetism and the Chemical Bond. John B. Goodenough. Interscience (Wiley), New York, 1963. xvi + 394 pp. Illus. $12.50. Science 143, 33 (1964).

[b34] Evans R. F. L. . Atomistic spin model simulations of magnetic nanomaterials. Journal of Physics: Condensed Matter 26, 103202 (2014). 24552692 10.1088/0953-8984/26/10/103202

[b35] Yanes R. . Effective anisotropies and energy barriers of magnetic nanoparticles with N\‘eel surface anisotropy. Physical Review B 76, 064416 (2007).

[b36] Lucas G., Burdet P., Cantoni M. & Hébert C. Multivariate statistical analysis as a tool for the segmentation of 3D spectral data. Micron 52–53, 49 (2013). 10.1016/j.micron.2013.08.005 24035679

[b37] Uhl M. & Siberchicot B. A first-principles study of exchange integrals in magnetite. Journal of Physics: Condensed Matter 7, 4227 (1995).

[b38] Sawatzky G. A., Geertsma W. & Haas C. Magnetic interactions and covalency effects in mainly ionic compounds. Journal of Magnetism and Magnetic Materials 3, 37 (1976).

[b39] Momma K. & Izumi F. VESTA 3 for three-dimensional visualization of crystal, volumetric and morphology data. J Appl Crystallogr 44, 1272 (2011).

