# Supplementary material for: Origin of reduced magnetization and domain formation in small magnetite nanoparticles
Source: Sci Rep. 2017 Apr 10;7:45997. doi: 10.1038/srep45997 (PMC5385549; doi:10.1038/srep45997)
Supplement: Supplementary Information [file srep45997-s1.pdf]

Supplementary information for:

Origin of reduced magnetization and domain formation in small magnetite nanoparticles

Zlatko Nedelkoski[1], Demie Kepaptsoglou[2], Leonardo Lari[1], Tianlong Wen[3,4], Ryan A. Booth[3], Samuel D. Oberdick[3], Pedro L. Galindo[5], Quentin M. Ramasse[2], Richard F.L. Evans[1], Sara Majetich[3]* and Vlado K. Lazarov[1]*

1. Department of Physics, University of York, Heslington, York, YO10 5DD, UK
2. SuperSTEM, Sci-Tech Daresbury Campus, Daresbury, WA4 4AD, UK
3. Physics Department, Carnegie Mellon University, Pittsburgh, Pennsylvania 15213, USA
4. State Key Laboratory of Electronic Thin Films and Integrated Devices, University of Electronic Science and Technology of China, Chengdu, China
5. Department of Computer Science and Engineering, Universidad de Cádiz, 11510 Puerto Real, Spain.

Email: vlado.lazarov@york.ac.uk; sara@cmu.edu

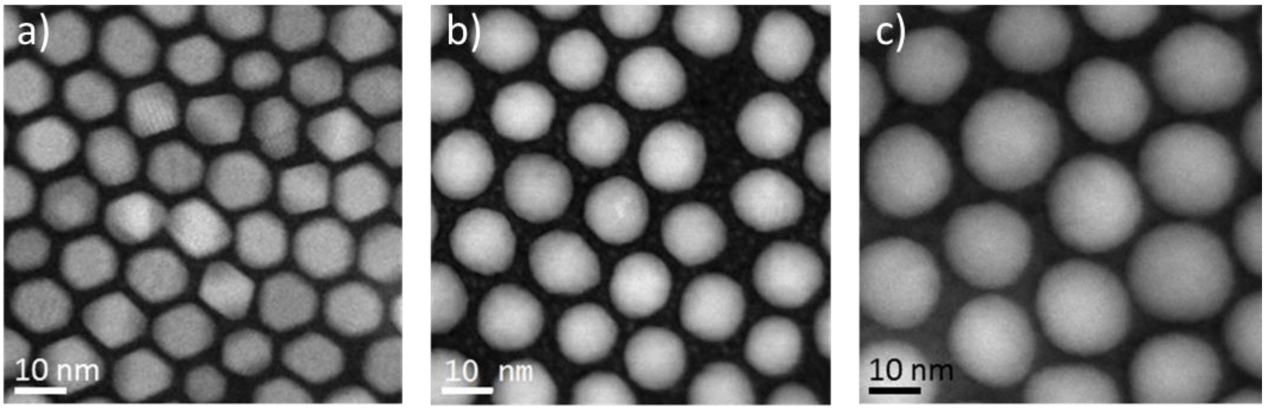

Supplementary Figure S1. Low magnification HAADF STEM images of a) Sun, b) Colvin, c) Hyeon NPs.

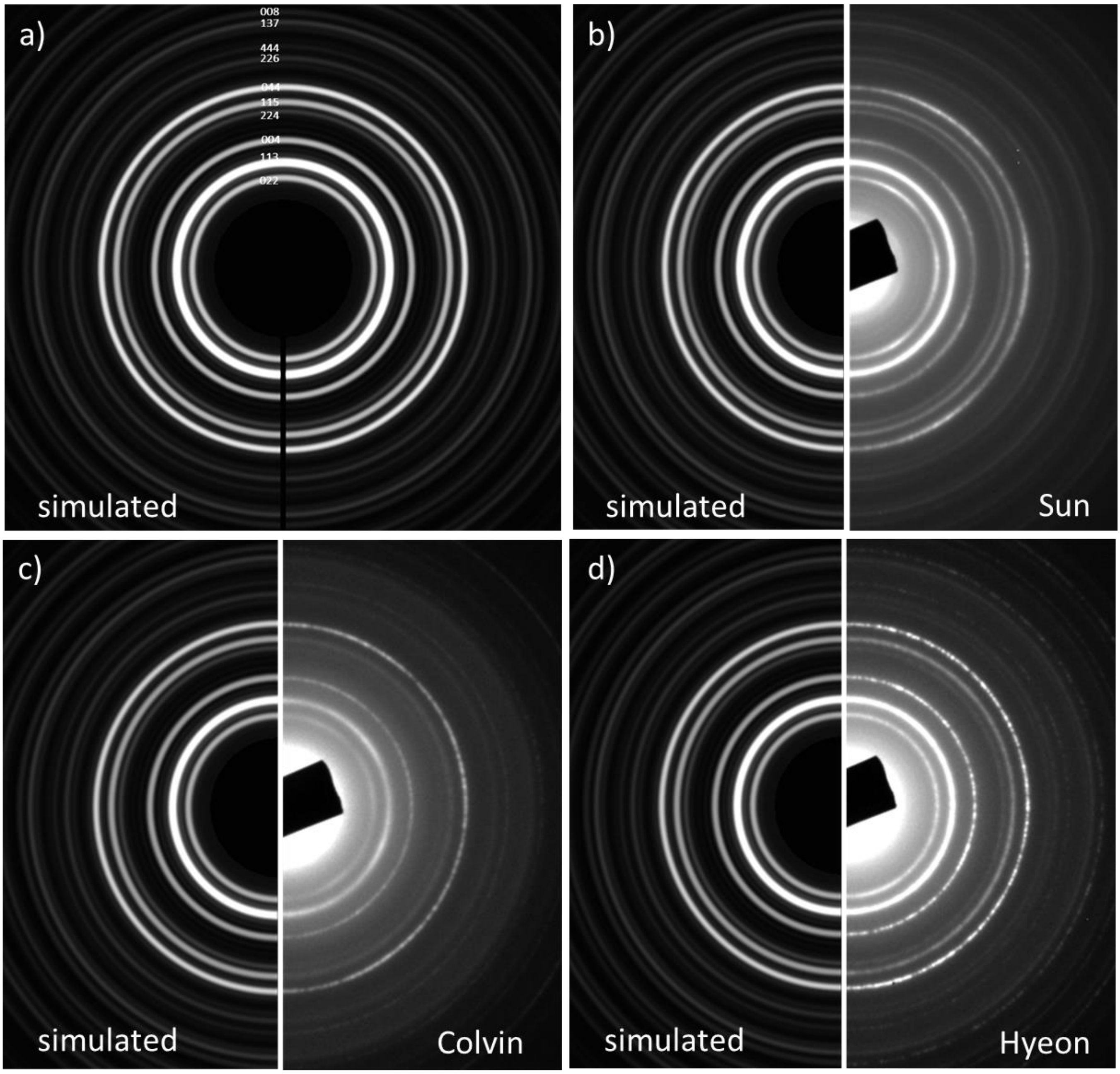

Supplementary Figure S2. a) Simulated SAED pattern for magnetite crystal structure. Experimental SAED patterns (right-hand side subfigures) of b) Sun, c) Colvin, d) Hyeon NPs compared with the simulated (from (a)) diffraction pattern (left-hand side subfigures in (b)-(d)). The SAED indexing is shown in (a).

Supplementary EELS discussion

Atomically resolved HAADF STEM imaging of individual NPs indicates that all three sets NPs are uniform without a visible core-shell structure; in all three cases the bulk like magnetite structural ordering extends all the way to the particle surfaces. The chemistry of selected individual NPs was further investigated by electron energy loss spectroscopy (EELS) measurements and more specifically the near-edge fine structures (ELNES) of the O *K* and Fe $L_{2,3}$ ionisation edges. Distinguishing between some of the different Fe oxide structures, for example Fe_3O_4 vs Fe_2O_3, can be challenging, as the differences in the O *K* and Fe $L_{2,3}$ ELNES between Fe_3O_4 and Fe_2O_3 are subtle. Arguably, the most distinctive difference lies in the intensity and position in energy of the peak labelled 'c' in the O *K* ELNES (**Supplementary Fig. S3**); this is typically accompanied by a change in intensity ratio between the Fe L_2 and L_3 edge peaks (**Supplementary Fig. S3**). For this reason O *K* and Fe $L_{2,3}$ EEL spectra acquired for the three different type NPs (**Supplementary Fig. S3**) are compared to that of a standard Fe_3O_4 sample acquired in identical conditions. The presented data are averaged from a large number of spectra extracted from 2D EELS spectrum images (**Supplementary Fig. S4, S5, S6)**, without any other signal processing apart from subtraction of the decaying background using a power law model. The measurements (repeated in several NPs in each of the three sets) showed no appreciable change in the Fe $L_{2,3}$ white-line intensity ratios, or shift of the edge onset across the observed nanoparticles, which would have been indicative of a Fe valence change. The similarities between all datasets confirms the initial conclusions from HAADF image observations about the chemical uniformity of the particles and the absence of a core-shell structure.

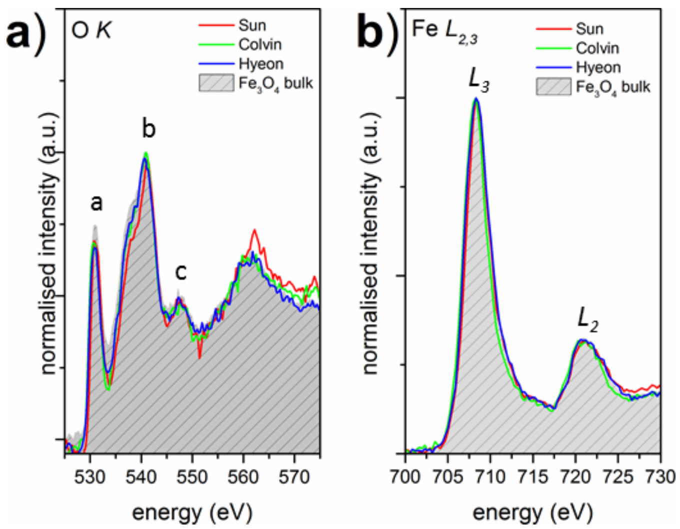

Supplementary Figure S3. Background subtracted EELS spectra (raw data) from the three sets of nanoparticles showing the a) O *K* and b) Fe $L_{2,3}$ edge from the three particle types, plotted along with spectra from a known bulk Fe_3O_4 sample.

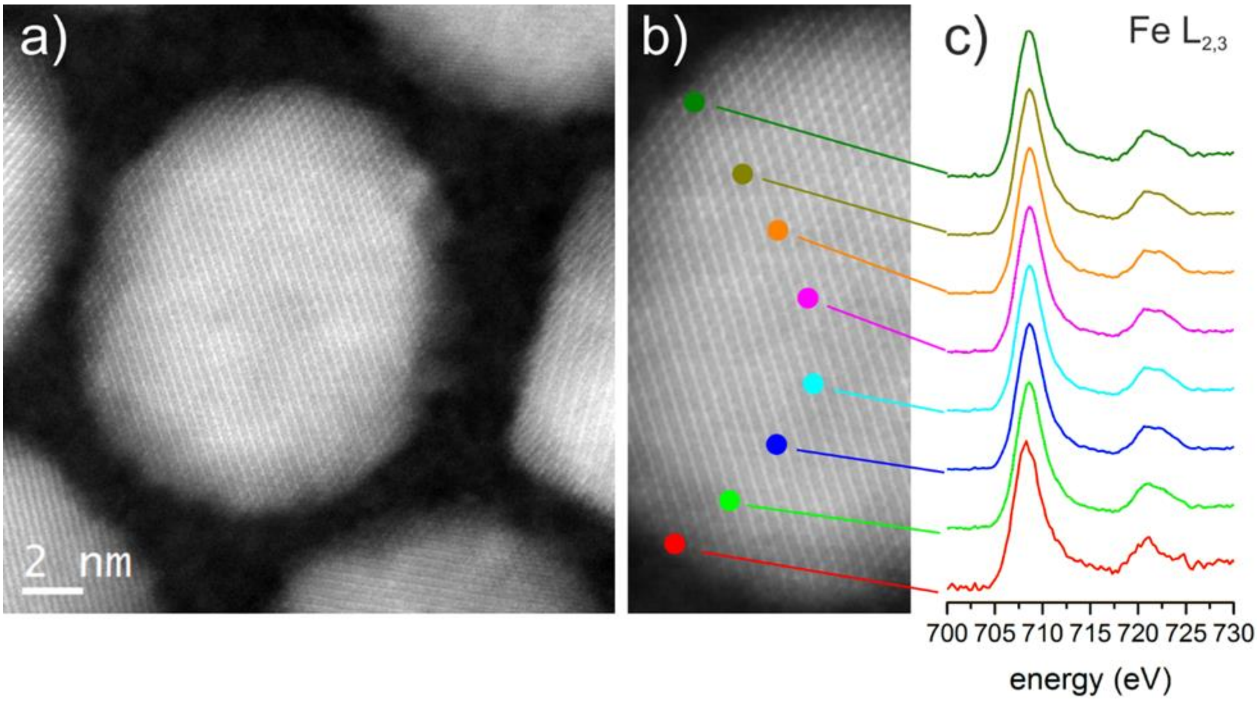

Supplementary Figure S4. a) HAADF STEM image of a Sun NP, b) HAADF STEM signal acquired simultaneously with the EELS 2D spectrum image, c) a number of Fe $L_{2,3}$ spectra are extracted from the spectrum image at the positions marked in (b).

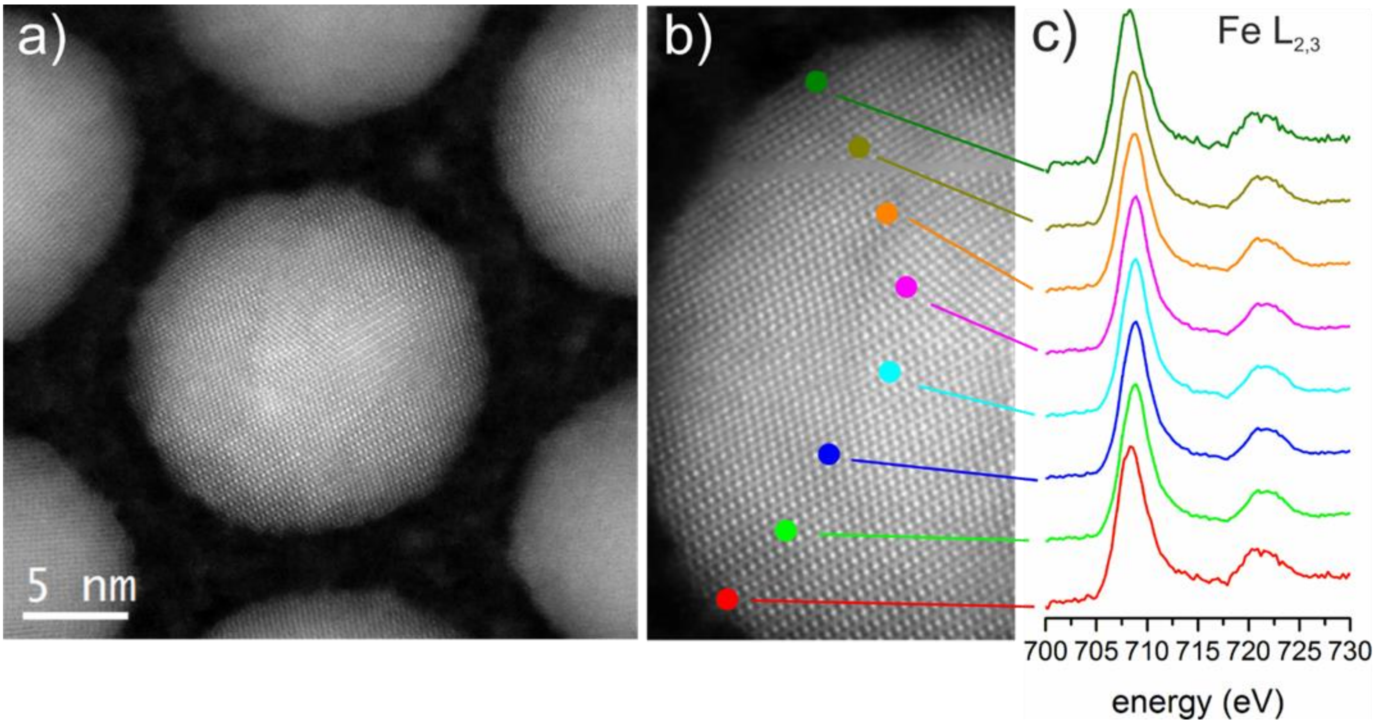

Supplementary Figure S5. a) HAADF STEM image of a Colvin NP, b) HAADF STEM signal acquired simultaneously with the EELS 2D spectrum image, c) a number of Fe $L_{2,3}$ spectra are extracted from the spectrum image at the positions marked in (b).

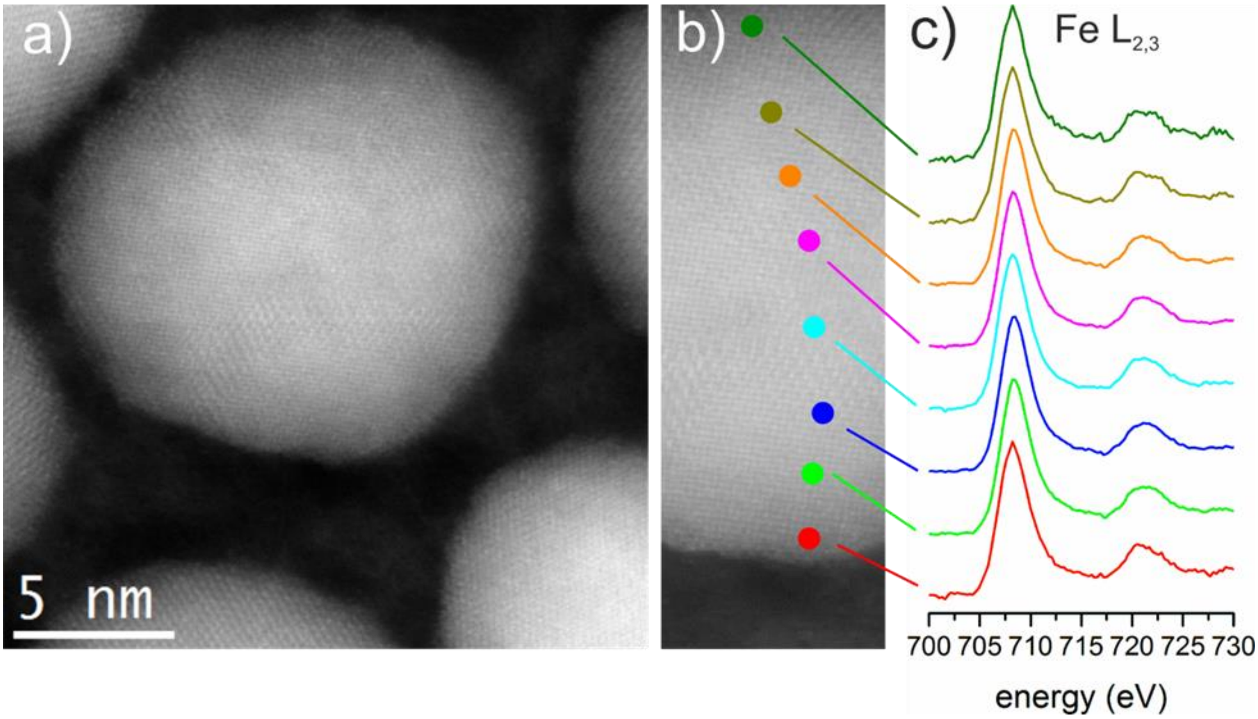

Supplementary Figure S6. a) HAADF STEM image of a Hyeon NP, b) HAADF STEM signal acquired simultaneously with the EELS 2D spectrum image, c) a number of Fe $L_{2,3}$ spectra are extracted from the spectrum image at the positions marked in (b).

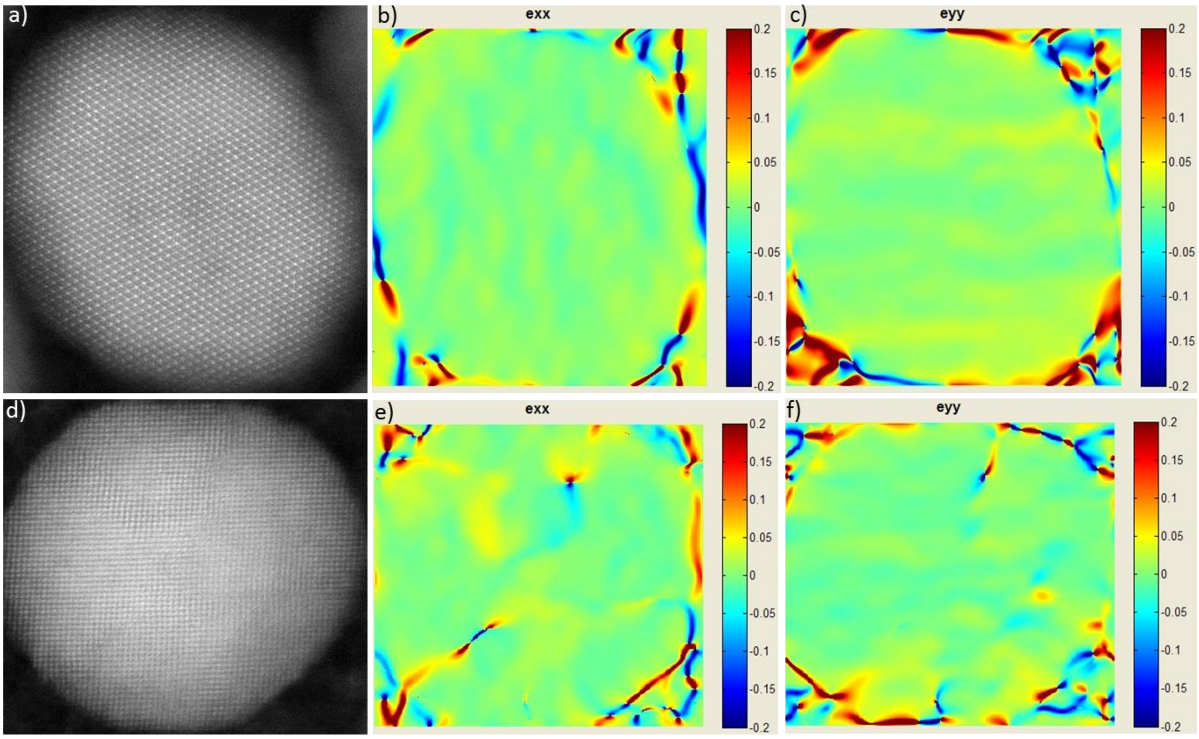

Supplementary Figure S7. a) HAADF STEM of a Sun particle; b) e_{xx} and c) e_{yy} strain maps for the particle shown in (a). d) HAADF STEM of a Colvin particle; e) e_{xx} and f) e_{yy} strain maps for the particle shown in (d). Strain maps were produced using the geometric phase analysis (GPA) method. Sun particles have no strain across the entire particle, while Colvin particles show strain in the vicinity of the defects.

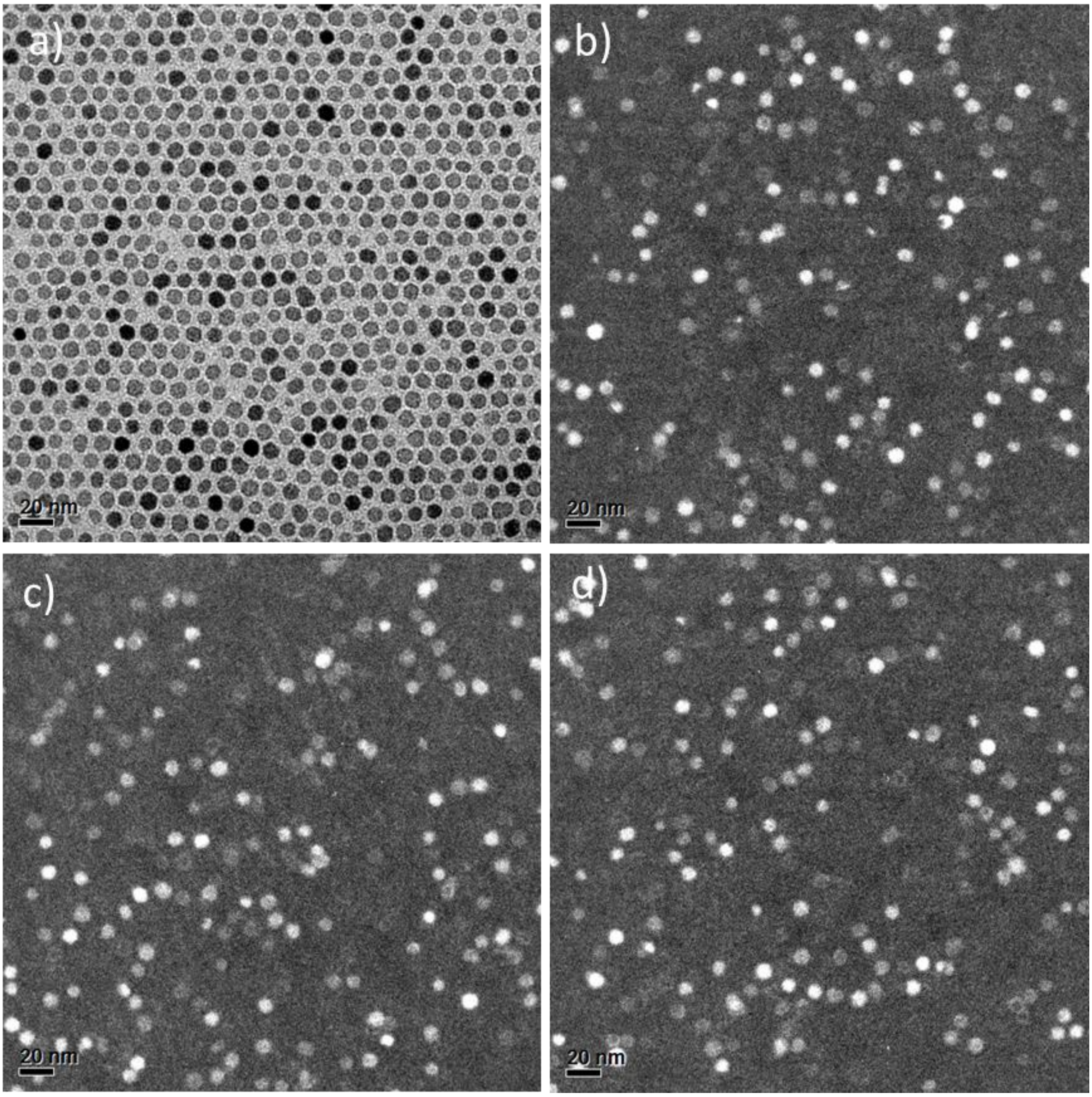

Supplementary Figure S8. Sun NPs. Low magnification a) bright field TEM, b)-d) dark field TEM images recorded using **g** = <220> from different positions along the corresponding diffraction ring.

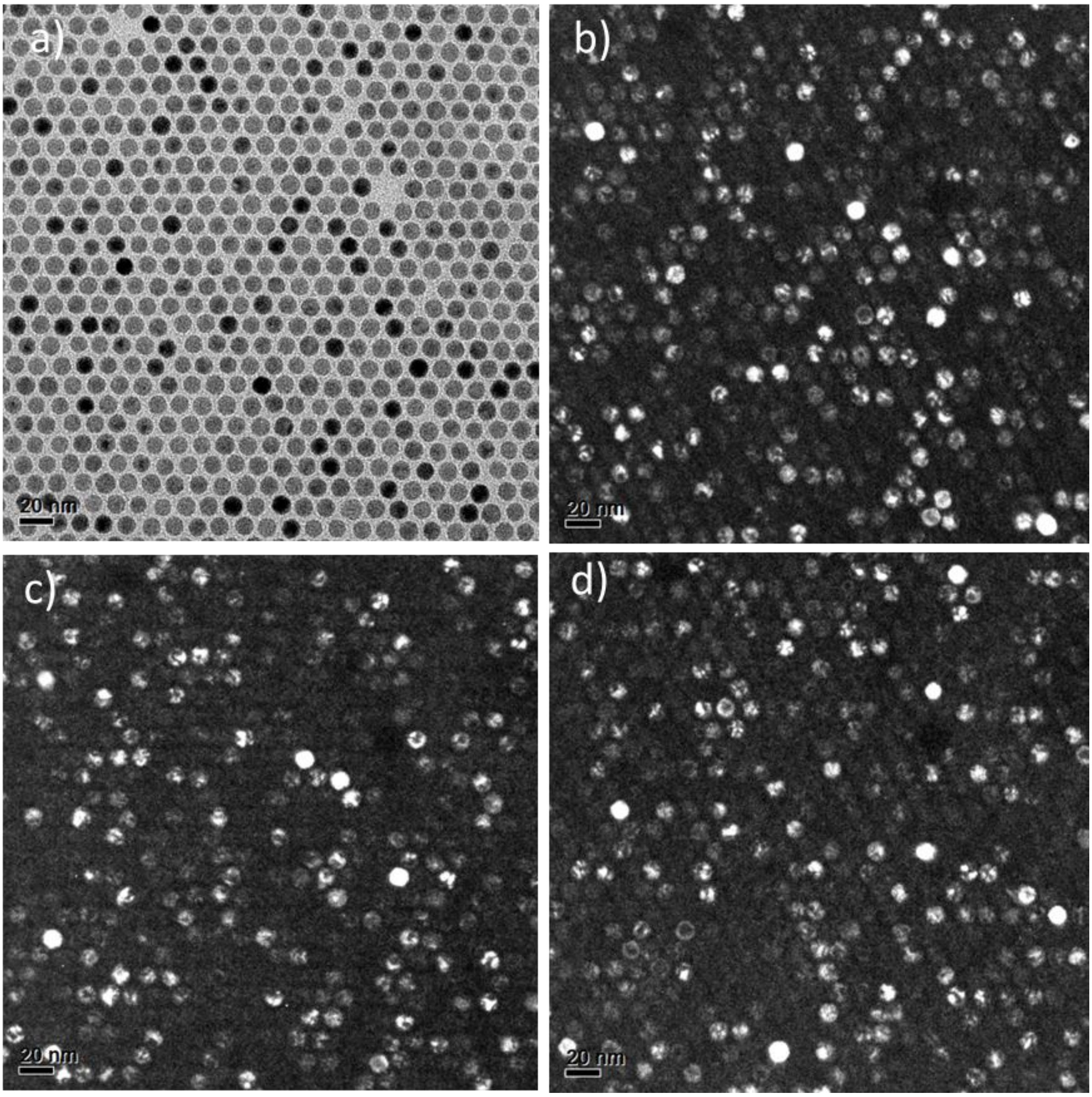

Supplementary Figure S9. Colvin NPs. Low magnification a) bright field TEM, b)-d) dark field TEM images recorded using **g** = <220> from different positions along the corresponding diffraction ring. In contrast to the Sun NPs, the Colvin NPs show abundance of structural defects distinguished by the dark contrast appearing along a defect boundary.

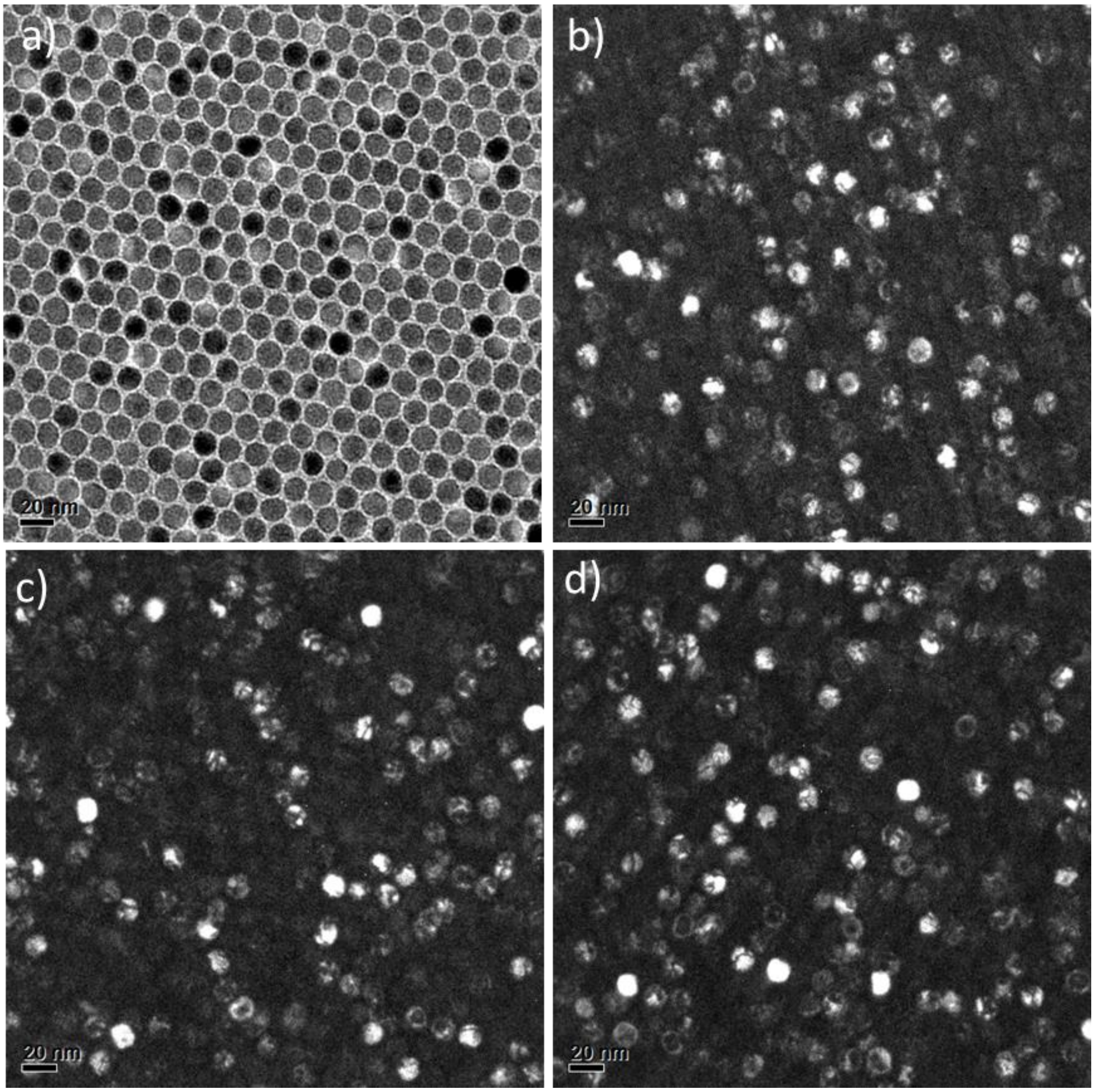

Supplementary Figure S10. Hyeon NPs. Low magnification a) bright field TEM, b)-d) dark field TEM images recorded using **g** = <220> from different positions along the corresponding diffraction ring. In contrast to the Sun NPs, the Hyeon NPs show abundance of structural defects distinguished by the dark contrast appearing along a defect boundary.

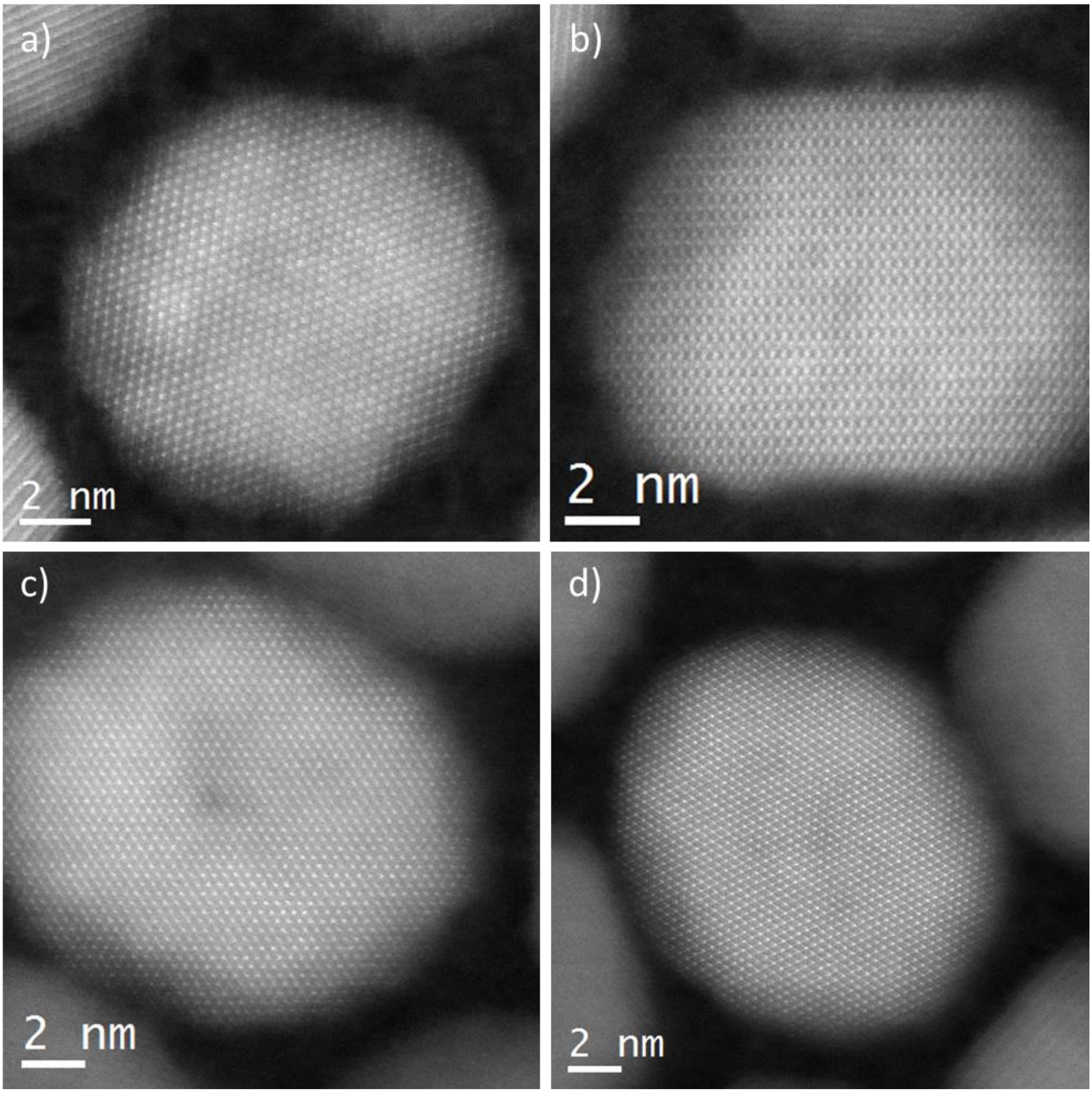

Supplementary Figure S11. Gallery of Sun NPs imaged in HAADF STEM mode.

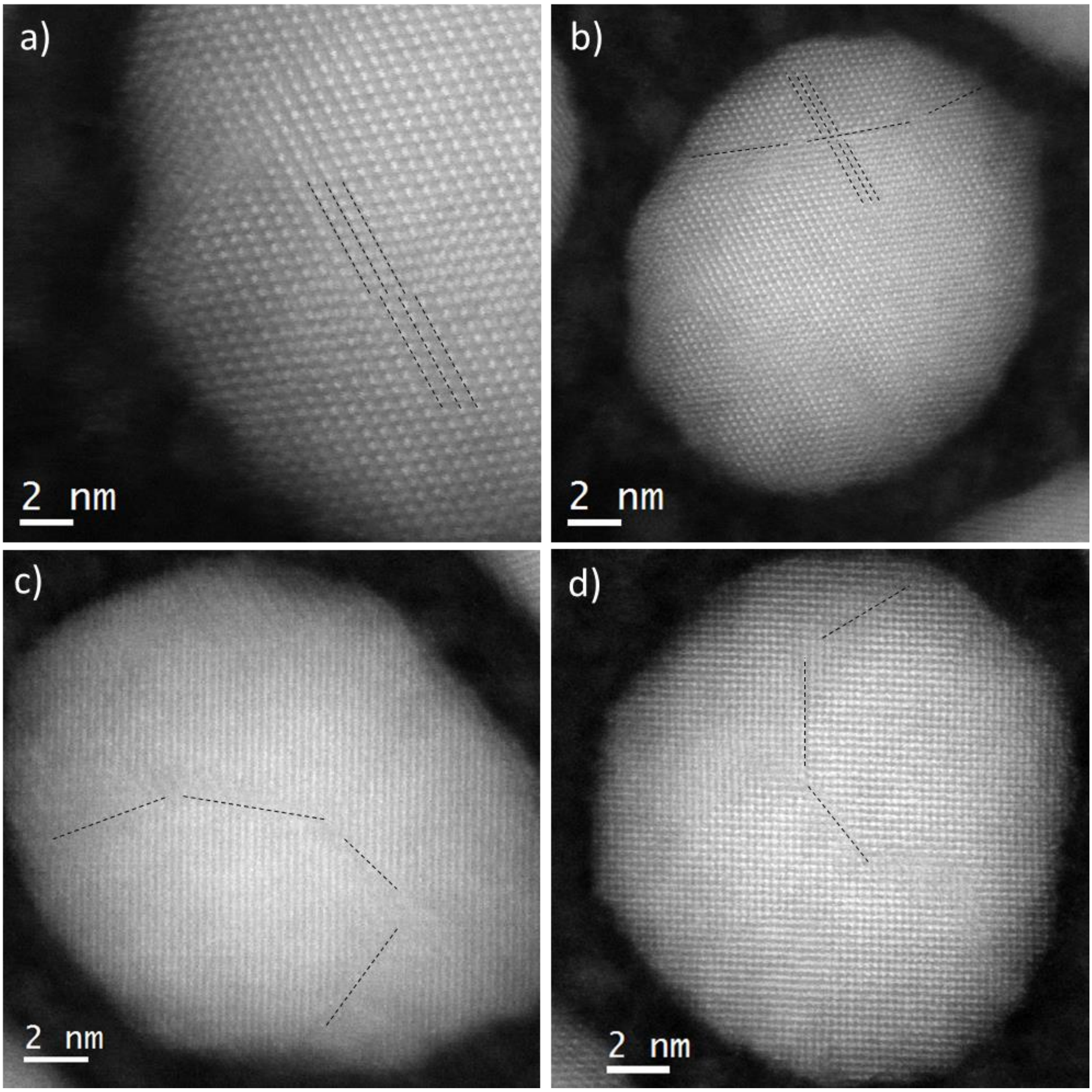

Supplementary Figure S12. Gallery of Colvin NPs imaged in HAADF STEM mode. The defects are outlined with the dashed lines.

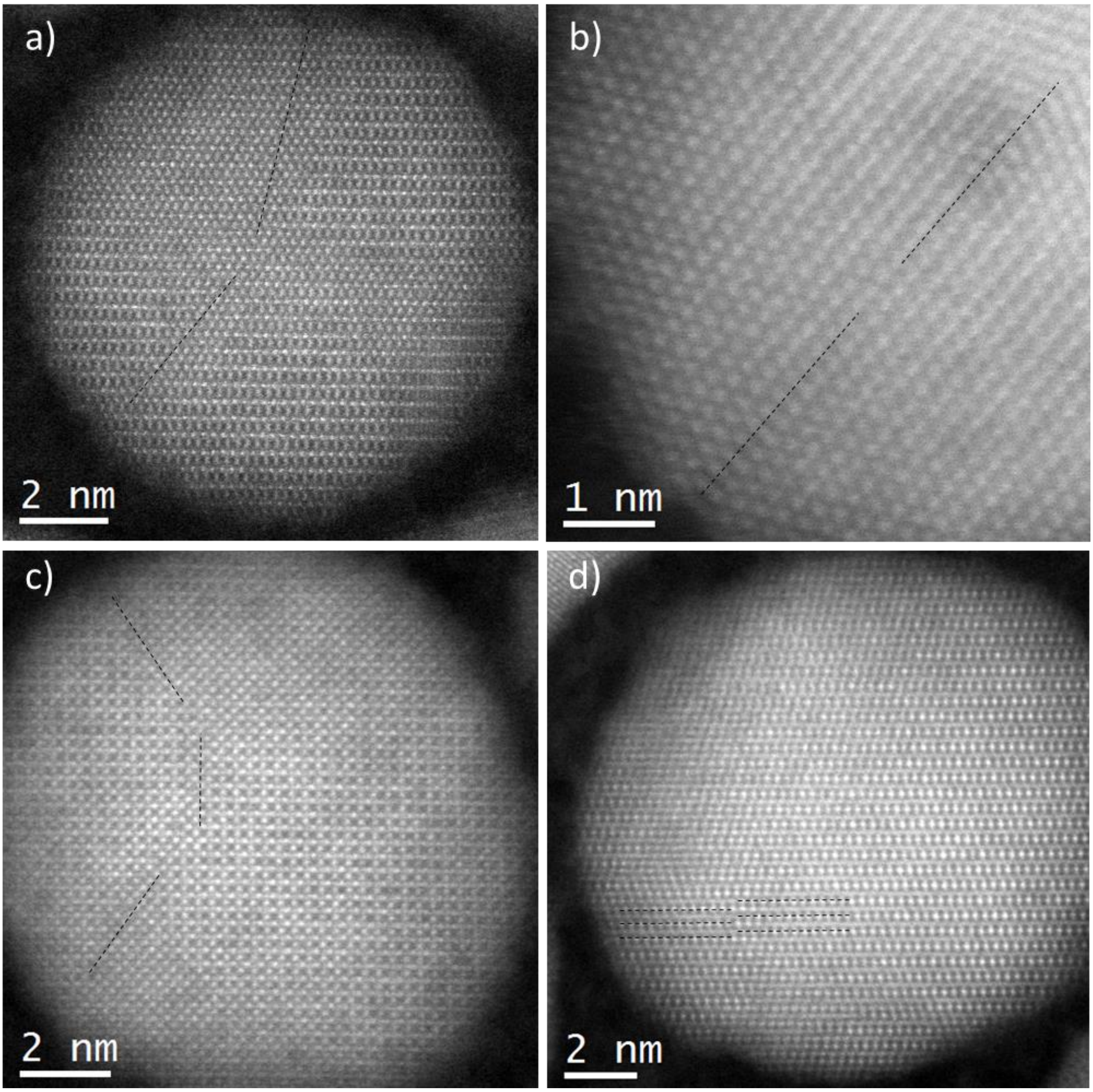

Supplementary Figure S13. Gallery of Hyeon NPs imaged in HAADF STEM mode. The defects are outlined with the dashed lines.

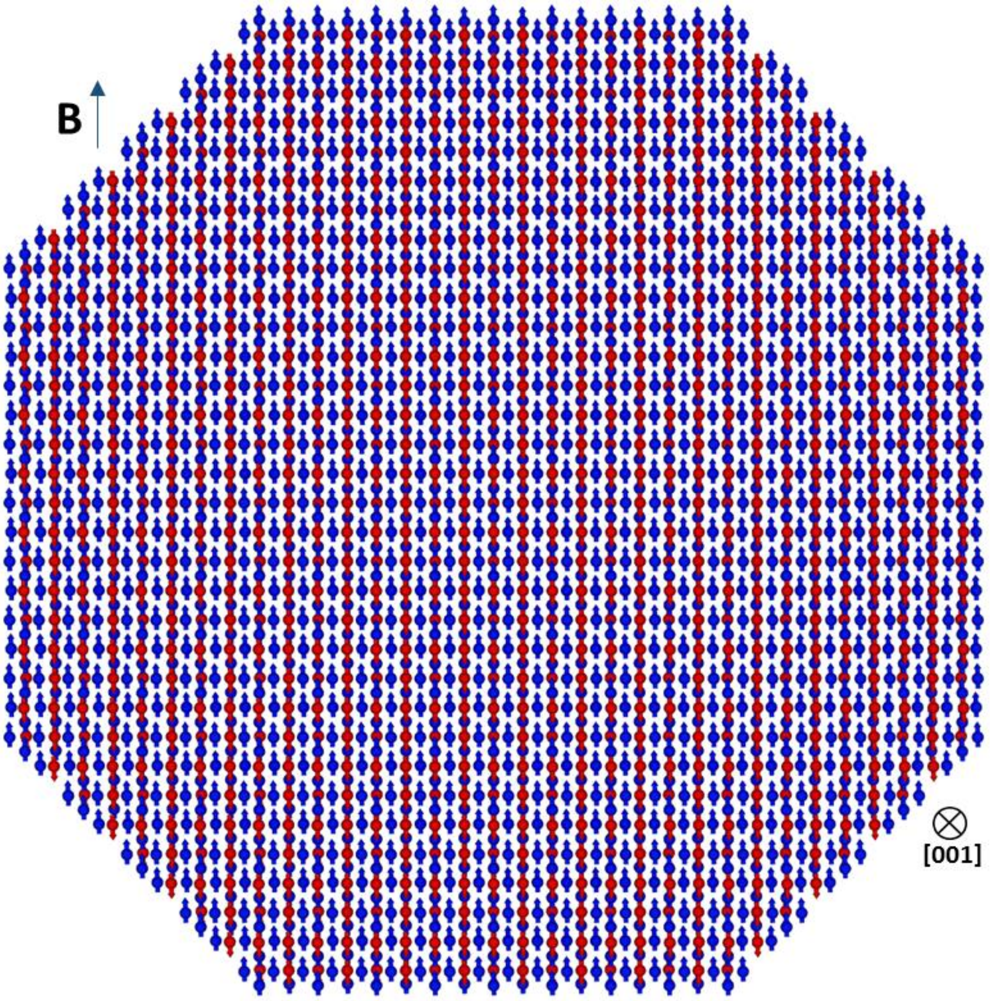

Supplementary Figure S14. Spin configurations at magnetic saturation (5T field) for faceted nanoparticle, showing that the faceting does not affect the magnetization. The nanoparticle is viewed along the [001] zone axis. Colour coding is the same as in Figure 4.

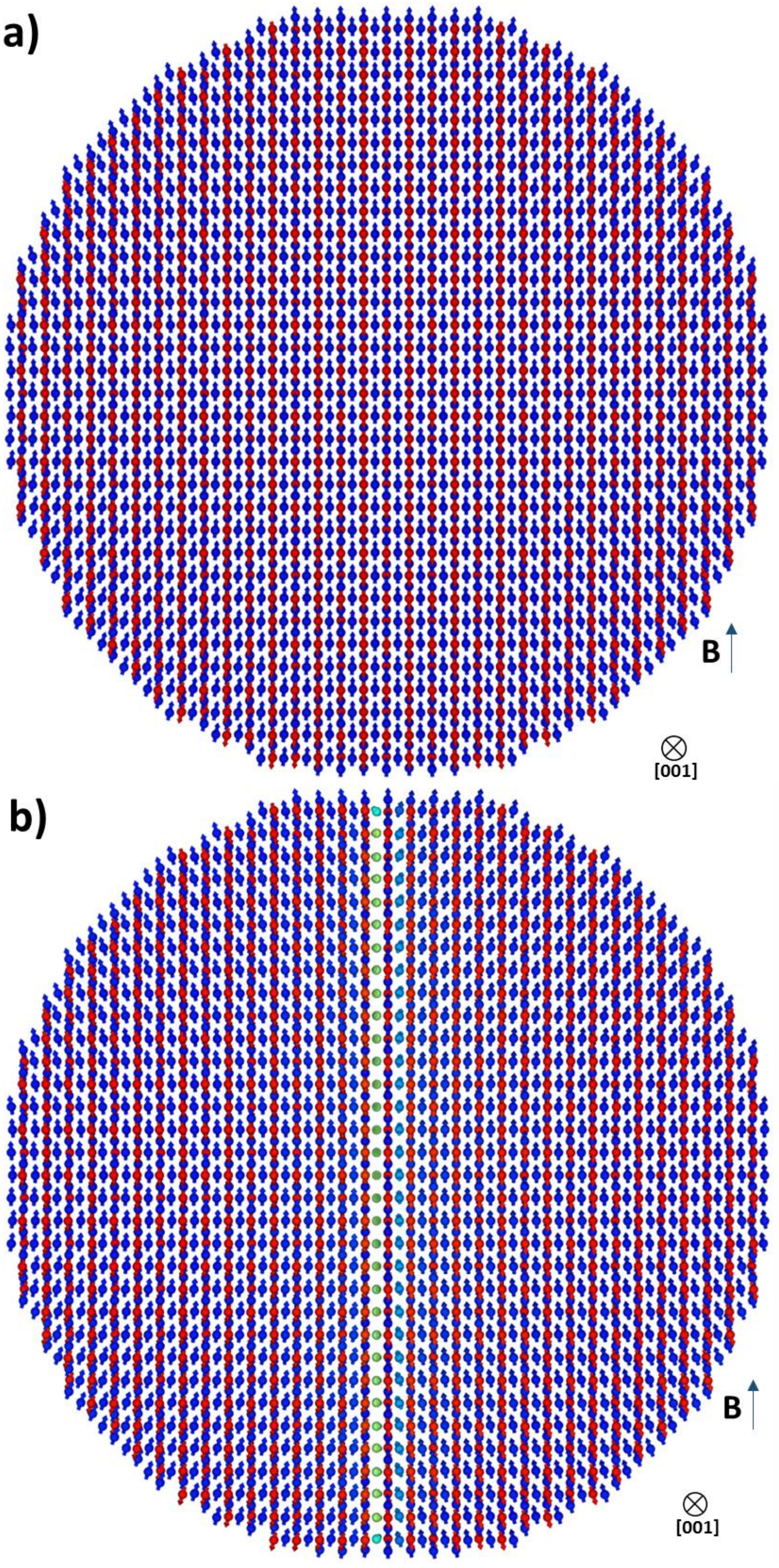

Supplementary Figure S15. Visualisations of the magnetic spin structure considering large surface anisotropy constant $-K_S/J = 1/8$ for a) a single crystal particle b) a particle with an APB. The strong surface anisotropy changes slightly the surface spin structure of the particles. We expect that in fact the surface anisotropy is weak, but these simulations show that even in the case of strong anisotropy the multidomain state caused by the APB is still present and quite stable.
